# Molecular detection and characterization of resistant genes in *Mycobacterium tuberculosis* complex from DNA isolated from tuberculosis patients in the Eastern Cape province South Africa

**DOI:** 10.1186/1471-2334-14-479

**Published:** 2014-09-04

**Authors:** Nolwazi L Bhembe, Uchechukwu U Nwodo, Sharlene Govender, Cindy Hayes, Roland N Ndip, Anthony I Okoh, Ezekiel Green

**Affiliations:** Department of Biochemistry and Microbiology, Faculty of Science and Agriculture, University of Fort Hare, PMB X1314 Alice, 5700 South Africa; Department of Microbiology and Parasitology, Faculty of Science, University of Buea, PO Box 63, Buea, Cameroon; Biochemistry and Microbiology, Nelson Mandela Metropolitan University, Summerstrand Campus (South), Port Elizabeth, South Africa; National Health Laboratory Services, Tuberculosis Section, Buckingham Road, Port Elizabeth, South Africa

**Keywords:** *Mycobacterium tuberculosis* complex, Tuberculosis, Multidrug resistance, Extensive drug resistance

## Abstract

**Background:**

Tuberculosis (TB) in both animals and humans is caused by *Mycobacterium tuberculosis* complex (MTBC) primarily transmitted by inhalation of aerosolized droplets containing the organism. Multi-drug resistance (MDR) and extensive drug resistance (XDR) are evolutionary features of *Mycobacterium tuberculosis* to subvert the antibiotic regimes in place. The heavy burden of TB worsened by HIV endemic in South Africa motivated for the investigation of MTBC prevalence among TB patients in Port Elizabeth and the amplification and sequencing of the DNA amplicons known to confer resistance to TB drugs.

**Methods:**

Three thousand eight hundred and ten (3810) sputum specimens were processed and DNA was isolated from sputum specimens collected from different hospitals and health care places in the Eastern Cape Province, South Africa. DNA was amplified using the Seeplex^®^ MTB Nested ACE detection assay. The agar-dilution proportion method was used to perform drug-sensitivity testing using 7H10 Middlebrook medium. Target genes known to confer resistance to first and second-line drugs were amplified and the amplicons sequenced.

**Results:**

One hundred and ninety (5%) DNA samples tested positive for MTBC and from the resistant profiles of the 190 positive samples, we noted that multidrug-resistant TB was identified in 189 (99.5%) with 190 (100%) patients infected with MTB resistant to isoniazid and 189 (99.5%) having MTB resistant to rifampicin. Other percentages of drug resistance observed including 40% pre-XDR and 60% of XDR.

**Conclusion:**

This study provides valuable data on the different kinds of mutations occurring at various target loci in resistant MTBC strains isolated from samples obtained from the Eastern Cape Province. The results obtained reveal a high incidence of MDR amongst the positive samples from Eastern Cape Province, South Africa.

**Electronic supplementary material:**

The online version of this article (doi:10.1186/1471-2334-14-479) contains supplementary material, which is available to authorized users.

## Background

The *Mycobacterium tuberculosis* Complex (MTBC) consists of nine bacterial species that cause tuberculosis (TB) in mammals, including human beings [1]. MTBC results in substantial economic losses in cattle herds and humans as it is usually found in the more economically active humans [2]. Tuberculosis is a major public health concern and a third of the world’s population is infected with some members of MTBC [1]. South Africa is a country with high incidence of TB, there were 550 cases per 100 000 population in 2003, 718 case per 100 000 population in 2004 [3, 4] and 600 cases per 100 000 population in 2005 [5]. The country had one of the worst recorded epidemics in the world in 2008 caused by the rising rates of HIV and the emergence of multidrug resistant TB [6]. The country is divided into nine provinces and among them the Eastern Cape has 80% of TB cases in South Africa [7]. The Eastern Cape is one of the poorest Provinces in South Africa and because of its poverty the spread of TB is enhanced [8]. Most people take TB as a disease of the past decade caused by strains that cannot be treated with existing drugs; this disease has turned to be one of the world’s most pressing health problems [6].

Resistance of the organisms to TB drugs is a major public health problem that threatens the progress made in TB control worldwide. Drug resistance arises due to improper use of antibiotics in chemotherapy of drug susceptible organisms [6]. Multidrug-resistance TB (MDR-TB) is resistant to the two most commonly used drugs (isoniazid and rifampicin) in the common four drug regimen [9]. In 2010, the World Health Organization (WHO) estimated that there were globally 290 000 cases of MDR-TB among cases of pulmonary TB that were reported [10]. There have been 1.8% increases in MDR-TB cases in South Africa. There are several factors that contribute to the development of MDR-TB, such as poor adherence of patients to first line anti-TB drugs, dosage and duration of treatment, inappropriate treatment regiments and non-compliance to national guidelines and TB protocol by TB clinicians [11].

Inappropriate use of second line drugs used in the treatment of TB leads to amplification of resistance and development of XDR-TB [10]. Extensively drug resistance TB (XDR-TB) is the TB resistant to any fluoroquinolone and at least one of the injectable drugs (capreomycin, kanamycin and amikacin) in addition to isoniazid and rifampicin. Several methods have been used to identify MTBC, including culture and biochemical tests such as acid-fast smears and sputum cultures. The diagnosis of TB includes history, physical examination and radiological findings in lung apices. Acid-fast smears and cultures of sputum are also required [12]. This study aimed at using a multiplex PCR targeting two genes (*mpb64* coding for immunogenic secretory protein specific for *Mycobacterium tuberculosis* complex and IS*6110* insertion element found exclusively within the members of the MTBC) for the detection of MTBC in sputum samples obtained from Eastern Cape Province and determination drug resistance using sequencing of the DNA amplicons known to confer resistance to TB drugs.

## Methods

### Sample collection

Three thousand eight hundred and ten sputum specimens in the Eastern Cape Province were collected from different hospitals and clinics from patients that showed clinical signs of TB and transported to the microbiology laboratory at Fort Hare University, South Africa for culture over a period of 24 months from January 2012 to 2013 December. Biodata including age (0–20; 21–40; 41–60 and 60 years and above) and gender of the patients were also collected.

### Bacteriological procedure

The bacteriological procedure was done as outlined by Balows et al. [13]. NaOH (4%) was used to kill any other contaminants in this procedure. Two Lowenstein-Jensen (LJ) slants were inoculated and incubated at 37°C for 6–8 weeks. A smear was prepared from each of the processed samples on a grease-free slide and stained by carbol fuschin using the Ziehl_Neelsen technique. Slides were checked for AFB under a microscope. Mycobateria were isolated from sputum. Isolation and identification of mycobacteria was carried out by the Microbiology service of each hospital using acid-fast staining (AFB).

### Drug susceptibility

Antibiotic susceptibility profiles were done at the National Health Laboratory Services in Port Elizabeth. Several spade-full of growth were scraped from LJ slants, transferred to a sterile screw-cap tube containing glass beads and 3 ml normal saline (0.85%) and mixed well on a vortex mixer. Turbidity was matched against McFarland standard no. 1. Inoculum (100 μl) was added to each plate, containing 5 ml 7H10 Middlebrook medium with drug in each quadrant. *M. tuberculosis* strain H37Rv was used as control in all sets of experiments. The inoculated plates were incubated at 37°C in an atmosphere of 10% CO_2_.

The agar-dilution proportion method was performed according to Balows et al. [13]. Lyophilized drugs were reconstituted aseptically in water. The stock was diluted in such a manner that a 5 μl aliquot contained the requisite amount of each drug. The drug concentrations used in this study were isoniazid (1 μg ml^-1^) Rifampicin (5 μg ml^-1^), Streptomycin (10 μg ml^-1^), Ethambutol (10 μg ml^-1^), Ethionamide (5 μg ml^-1^), ofloxacin (2 μg ml^-1^), Amikacin (6 μg ml^-1^) and capreomycin (40 μg ml^-1^). Results were recorded after 3 weeks. Each drug-sensitivity test was carried out at least three times and the average was recorded.

### Identification of MTBC species using Seeplex^®^MTB nested ACE detection assay

The Seeplex^®^ MTB Nested ACE detection assay (Seegene Inc, Korea) was carried according to the manufacturer’s instructions using a thermal cycler (Bio-Rad, South Africa). The assay is a multiplex PCR involving the first PCR (1 cycle at 94°C for 15 min; 40 cycles at 94°C for 30 s, 60°C for 30 s, 72°C for 30 s; 1 cycle at 72°C for 5 min) and a nested PCR (1 cycle at 94°C for 15 min; 30 cycles at 94°C for 30 s, 62°C for 30 s, 72°C for 30 s; 1 cycle at 72°C for 5 min). The amplicons were separated on 2% agarose gel electrophoresis, at 100 V for 90 minutes using TBE buffer pH 8.3. The gel was thereafter visualized under Alliance 4.7 transilluminator (UVITEC Limited, Cambridge, UK).

### ***Kat*G,*rpoB*,*rrs*and*eis*gene amplification through Polymerase Chain Reaction**

Resistant genes to first-line drugs were amplified using primers RTB511f (5′ TGGCACGCTGCCGGCACCTA) and RTB 311r (5′ CGAAGCCGAACCCGAACGTC) for *kat*G gene. After initial denaturing at 93°C for 5 min, 3-step cycling for 30 amplification cycles were completed each consisting of 1 min at 95°C, 1 min at 64°C and 2 min at 72°C. A final extension of 10 min at 72°C was applied. For the *rpo*B gene the RDRSf (5′GTCGGTCATGTTCGCGATCG) and RDRAr (5′ TCGGCCAGGTAGTCGCTGAT) primers were used. After initial denaturing at 95°C for 5 min, 3-step cycling for 40 amplification cycles were completed each consisting of 1 min at 95°C, 1 min at 64°C and 1 min at 72°C. A final extension of 10 min at 72°C was applied. The molecular detection of resistant genes to second line drugs was done by amplifying the DNA with second line drugs primers; RRS2f (5′ TGCCGGGGTCAACTCGGAGG) and RRS2r (5′ GAACCCCTCACGGCCTACGC) for the *rrs* gene. After initial denaturing at 94°C for 4 min, 3-step cycling for 35 amplification cycles were completed each consisting of 1 min at 94°C, 1 min at 58°C and 2 min and 30 s at 72°C. A final extension of 10 min at 72°C was applied. Eisf1 (5′ GCCATGGGACCGGTACTTGC) and Eisr1 (5′ GTAGATGCCGCCCTCGCTAG) for the *eis* gene was the second primer used to amplify resistant genes to second line drugs with initial denaturing at 94°C for 4 min, 3-step cycling for 35 amplification cycles were completed each consisting of 1 min at 94°C, 1 min at 54°C and 2 min and 30 s at 72°C and a final extension of 10 min at 72°C.

The samples were amplified by PCR using the synthetic oligonucleotide primers that have been mentioned above. PCR was carried out in 25 μl tube containing 12.5 μl of master mix with (Tris pH 8.0, MgCl_2_, dNTP, 1UTaq polymerase), 8.5 μl water (DDW molecular grade), 1 μl of each primer and 2 μl of DNA template [14]. *Mycobacterium* H37RV strain was used as a positive control and sterile water used as a negative control. The amplified products were separated in 2% agarose gels together with 100 bp ladder (Fermentas), applied in a separate lane. Gels were stained with ethidium bromide and photographed on UV transiluminator.

### Sequencing of *KatG*, *rpoB*, *rrs* and *eis*genes

To check for resistance and possible mutations, twelve amplicons per gene of the amplified products were sequenced. DNA sequencing was performed using a Big Dye Terminator DNA sequencing kit v3.1 (Applied Biosystems, UK). Direct sequencing was done with 2 μL of chromosomal DNA, 0.25 μL of primer (10 pmol per μL), 2 μL of Big Dye buffer and 2 μL of Big Dye. Cycle parameters included a denaturation at 96°C for 10 s, annealing at 50°C for 20 s, and extension at 60°C for 4 min over 30 cycles, followed by Agencourt CleanSeq clean up. Sequences were determined by electrophoresis with the ABI 3130xl DNA sequencer (Applied Biosystems, UK). Editing of the sequences was performed using Bioedit Alignment Editor. Cleaned sequences were sent to BLAST using the nBLAST in NCBI (http://www.ncbi.nlm.nih.gov/); resistant genes were categorized to resistance types by their resistance profiles and sequence similarity [15]. The study was approved by the research ethics committee of the Goven Mbeki Research and Development Centre, University of Fort Hare, Record Number 2012/2013-01356. Written informed consent from participants was obtained and the confidentiality of the patients’ identities was protected.

## Results and discussion

### Amplification of Seeplex DNA samples results

**Figure 1:**
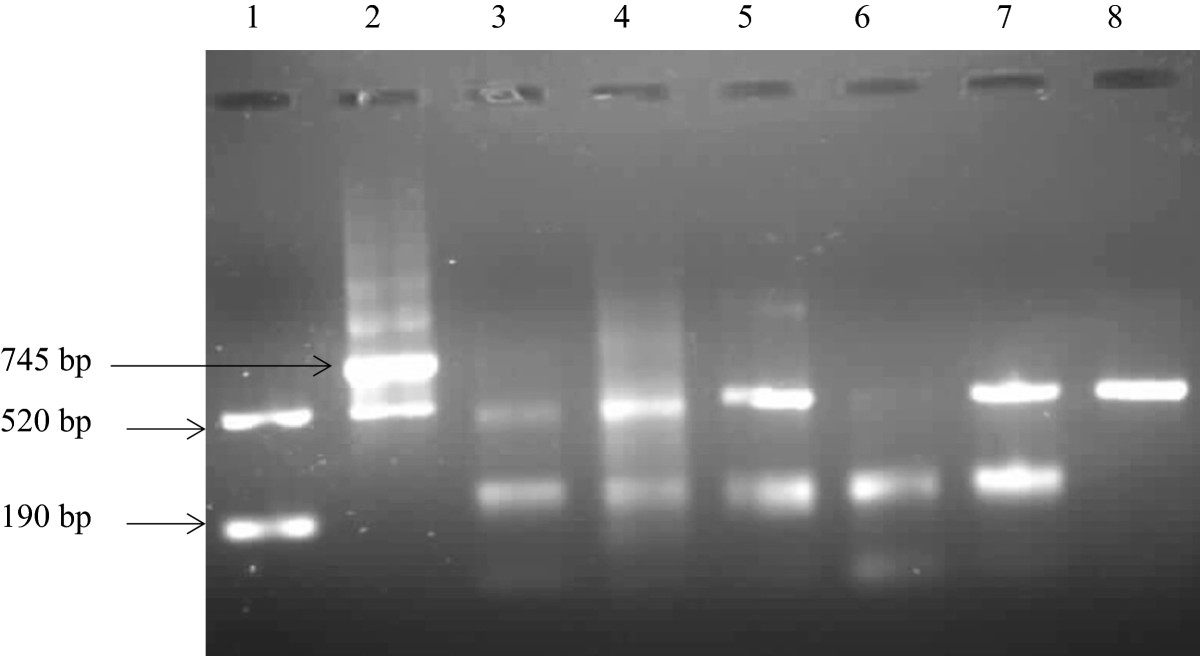
***Mycobacterium tuberculosis*** **complex detection through the amplification if the MPB64 immunogenic protein.** The DNA Marker shows two bands which are the internal control band (520 bp) and the *M. tuberculosis* band at (190 bp). This shows how positive samples should be. The positive control shows two bands as well, the internal control (520 bp) and an upper band corresponding to 745 bp (instead of 190 bp) which is designed by the manufacturer to eliminate false positive resulting from cross contamination. The negative control shows only the internal control (520 bp), negative samples shows only this band. Lane 1: DNA Marker; lane 2: Positive control; lane 3–7 DNA samples; lane 8: Negative control.

One hundred and ninety (190) DNA samples out of the three thousand eight hundred and ten (3810) specimens were used in the study. This is 5% of the specimens that were positive for MTBC. The Seeplex^®^ MTB Nested ACE detection assay target multi DNA regions (*mpb*64 and IS*6110*). The internal control (520 bp) is used to identify processed samples containing substances that may interfere with PCR amplification. The *M. tuberculosis* band corresponds to 190 bp and this is the band that confirms that this is *M. tuberculosis* complex (Figure 1). The main strength of the Seeplex^®^ MTB Nested ACE detection assay is that it uses multi-target PCR (IS*6110* and *mpb*64) for the specific detection of MTBC only; this prevents false positive results that are caused by other mycobacteria. However, the assay has a weakness of not differentiating amongst the different members of the MTBC. The sample in lane 6 showed a band at 190 bp and a very faint one at 520 bp, therefore we then re-ran a gel to be sure of the band and we properly mixed the DNA before loading to a 2% agarose gel and two visible bands were obtained at 190 bp and 520 bp.

From the biodata (Table 1) we observed that most of the patients were from the black race with 84.2% (160/190) and the rest were of the mixed race 15.8% (30/190). We then compared our findings with the population of the Nelson Mandela Bay Metropolitan Municipality (Port Elizabeth) which is 1 152 115 [7]. According to Census [7], 60.1% are black African, 23.6% mixed race, 14.4% white and 1.1% Indian/Asian. Of the population, 552 994 (48%) are male and 599 121 (52%) are female. “Young people (0–14 years) constitute 25, 5% of the population, youth (15–35 years) 37,1%, adults (36–64 years) 31,4% and the elderly (65+ years) 6%” [16].

**Table 1:** **Patient’s biodata**

Age (years)	Males N (%)	Females N (%)		Race	
			Blacks N (%)	Mixed race N (%)	Whites N (%)
**0–14**	2 (1.05)	7 (3.68)	6 (3.160)	3 (1.58)	0
**15–24**	8 (4.21)	24 (12.63)	28 (14.74)	4 (2.11)	0
**25–44**	52 (27.4)	65 (34.21)	100 (52.6)	17 (8.95)	0
**45–64**	19 (10)	9 (4.74)	22 (1.58)	6 (3.16)	0
**65+**	1 (0.53)	3 (1.58)	4 (2.11)	0	0
Total	82 (43.16)	108 (56.84)	160 (84.2)	30 (15.79)	0

N (%), number and percentage.

When looking at the statistics by Census [7], black Africans are more than any other race in this Municipality. This results show high prevalence of MTBC amongst the black race which could be due to the fact that these samples were collected from public clinics, where most black people frequently visit because of the low cost. Some mixed race people attend public clinics and some attend private clinics and that could be one of the reasons there was 15.8% mixed race that had MTBC. However it could also be because of sampling bias where only samples from eastern part of the Eastern Cape were received irrespective of gender or race. The mixed race and black people work together most of the time and some attend the same schools and stay in the same location which increases the risk of transmission of MTBC between the two races.

A fascinating observation from our results (Table 1) was made in this study where were observed that there are more females (56.8%) in this study that were detected to have MTBC in comparison to males (43.16%) which contradicts what other studies have reported on [6, 17]. It has been reported that in most of the world, more men than women are infected by MTBC [7]. A report by Census [7] gave the population of the people in Nelson Mandela Bay Metropolitan Municipality 552 994 (48%) are male and 599 121 (52%) are female which shows that there are more females than males in this region. However, our results concerning females being more infected by MTBC compared to males might be due to the fact that females care for the sick, both children and their husbands or brothers. It can also be as a result of selection bias where more samples for females were chosen than males.

Nevertheless most women die due to TB [6] and this affect woman mainly in their economically and reproductively active years [6]. This was shown in our study (Table 1) where 47% of women aged 15–44 years are the most infected. Our results are also in agreement with a study by Murray et al. [18] who found out that in women aged 15–44 years in developing countries TB is the most common cause of morbidity and mortality combined, and it kills more women than any other infectious disease including malaria and AIDS [18]. It was noted that the group from 15–44 years is a sexually and economically active age groups which could be another reason of having more females detected with MTBC. This does not mean that TB is contracted through sexual intercourse but it is transmitted by having a close contact with someone infected or an animal. This result (Table 1) indicates that TB can infect any race exposed to MTBC despite their genetic make-up and age group.

### Drug susceptibility results

One hundred and ninety (100%) of the patients were found to be resistant to at least one or more anti-TB drugs (Table 1). Resistance to only one drug was found in all 190 patients who were infected with MTBC resistant to isoniazid 190 (100%) as the highest resistance while the lowest resistant profile was observed on ethambutol with 9.5%. Almost all the samples (99.5%) that were resistant to isoniazid were also resistant to rifampicin. The findings of the study supports what other authors hypothesized which states that rifampicin can be used as a surrogate marker for MDR, this is due to the fact that 99.5% of rifampicin resistant *Mycobacterium tuberculosis* strains are equal to isoniazid [19–21]. Only one (0.53%) sample was susceptible to rifampicin. This is a first report of high drug resistant MTBC Port Elizabeth; these results are higher than those that were reported by Green et al. [22] who reported on 58.4% MDR-TB in the Mpumalanga Province of South Africa.

From the resistant profiles (Table 2) we noted that multidrug-resistant TB was identified in 189 (99.5%). Drug resistant TB develops from inadequate treatment of pulmonary TB [23]. Resistance to drugs used for TB treatment may also be due to poor drug selection by medical doctors [24]. Suggestion of several biological mechanisms linking drug-resistant TB and HIV has been made [25]. Drug malabsorption in HIV-infected patients, especially rifampicin and ethambutol can lead to drug resistance leading to treatment failure [23]. Malabsorption is caused by the damage to the intestinal villi caused by HIV, *Cryptosporidium*, one of the commoner and more serious opportunistic gut infections [26–28]. Possible mechanisms responsible for malabsorption HIV/AIDS include the impact of HIV on villi specitre enzyme deficiencies in intestinal mucosa, the opportunistic infections and altered intestinal transit have all been considered but these are mainly conjectural and effective treatments remain to be developed [29]. Data that supports this hypothetic statement has not yet been observed in humans [30]. Other studies done in South Africa also did not find association between HIV-infection and MDR-TB such as a retrospective study conducted in Durban, where 2.4% of 42 HIV co-infected and 11.5% of 253 HIV negative patients had MDR-TB [31]. In a study in Cape Town MDR-TB was 2.6% in 155 HIV negative in comparison of 32% in 93 HIV co-infected patients [32]. In gold miners, MDR-TB rate was 5.3% among 207 HIV co-infected and 6.5% of 218 HIV negative miners [33].

**Table 2:** **Susceptibility and resistance profile of** ***M. tuberculosis*** **genotypes to first and second line anti-mycobacterium drugs**

	TB resistance profiles to first line (FL) drugs					TB resistance profiles to second line (SL) drugs					
Parameter	INH	RIF	STM	EMB	MDR	ETHIO	OFL	CAP	AMIK	Pre-XDR	XDR
Age	N (%)	N (%)	N (%)	N (%)	N (%)	N (%)	N (%)	N (%)	N (%)	N (%)	N (%)
≤ 20	23 (12.1)	22 (11.6)	12 (6.3)	2 (1.1)	22 (11.6)	2 (1.1)	1 (0.53)	2 (1.1)	4 (2.1)	2 (1.1)	23 (12.1)
21–40	114 (60)	114 (60)	73 (38.4)	20 (10.5)	114 (60)	23 (12.1)	30 (15.8)	7 (3.6)	58 (30.5)	18 (9.4)	83 (43.7)
41–60	46 (24.2)	46 (24.2)	21 (11.1)	3 (1.58)	46 (24.2)	5 (2.63)	3 (1.6)	4 (2.1)	18 (9.5)	12 (6.3)	46 (24.2)
≥61	5 (2.6)	5 (2.6)	2 (1.1)	0 (0)	5 (2.6)	2 (1.1)	2 (1.1)	0 (0)	3 (1.6)	1 (0.53)	5 (2.6)
**Total**	**188 (98.9)**	**187 (98.4)**	**108 (56.8)**	**25 (12.7)**	**187 (98.4)**	**32 (16.8)**	**36 (18.9)**	**13 (6.8)**	**83 (43.7)**	**33 (17.4)**	**157 (82.6)**
**Gender**											
Male	85 (43)	84 (42.6)	47 (23.5)	8 (4)	84 (42.6)	17 (8.6)	14 (7.1)	5 (2.5)	36 (18.2)	17 (8.6)	85 (38)
Female	105 (55.3)	105 (55.3)	60 (31.6)	10 (5.3)	105 (55.3)	15 (7.9)	22 (11.6)	8 (4.21)	47 (24.7)	16 (8.42)	85 (44.7)
**Total**	**190 (100%)**	**189 (99.5)**	**107 (56.3)**	**18 (9.5)**	**189 (99.5)**	**32 (16.8)**	**36 (18.9)**	**13 (6.8)**	**83 (43.7)**	**33 (17.4)**	**157 (82.6)**

INH = isoniazid, RIF = rifampicin, STM = streptomycin; EMB = ethambutol; MDR = multi-resistant drug, ETHIO = ethionimide, OFL = ofloxacin, CAP = capreomycin, AMIK = amykacin, pre-XDR = pre-extensively drug resistant, XDR = extensively drug resistant.

On the second line drugs resistance to capreomycin was the lowest 13 (6.8%) and the highest was amykacin in 83 (43.7%). Pre-extensively drug tuberculosis was detected in 33 (17.4%) patients and extensively drug resistant TB was detected in 157 patients. Thirty three isolates (17.4%) were identified as pre-XDR and one hundred and fifty seven isolates were identified as XDR-TB. Comparing our results with the study by Campbell et al. [34] who reported ofloxacin (69: 21.9%) and amykacin (1:0.3%), our values are only high in amikacin resistance which was 43.7%. Of the tested isolates 55 (16%) were susceptible to all the study antibiotics and 10 (3%) were determined to be XDR- *M. tuberculosis*[34]. Our results show a high drug resistance of second line drugs in the Eastern Cape, South Africa.

### Mutation conferring resistance to INH, RIF, CAP and KAN drugs

**Figure 2:**
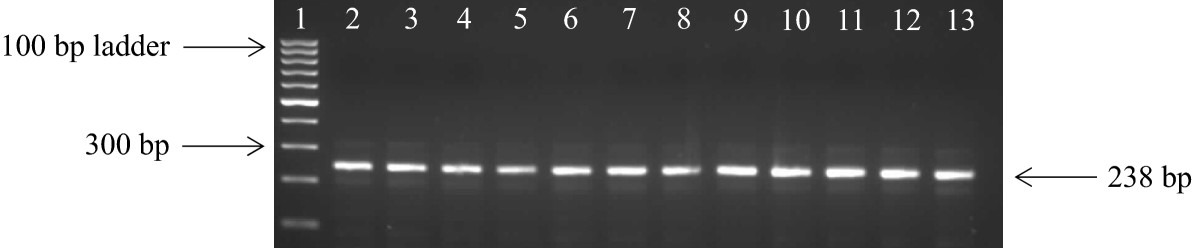
**Amplification of the** ***rpoB*** **gene in DNA samples.** Lane 1: 100 bp ladder; lane 2: positive control; lane 3–13: DNA isolates; lane 14: negative control.

**Figure 3:**
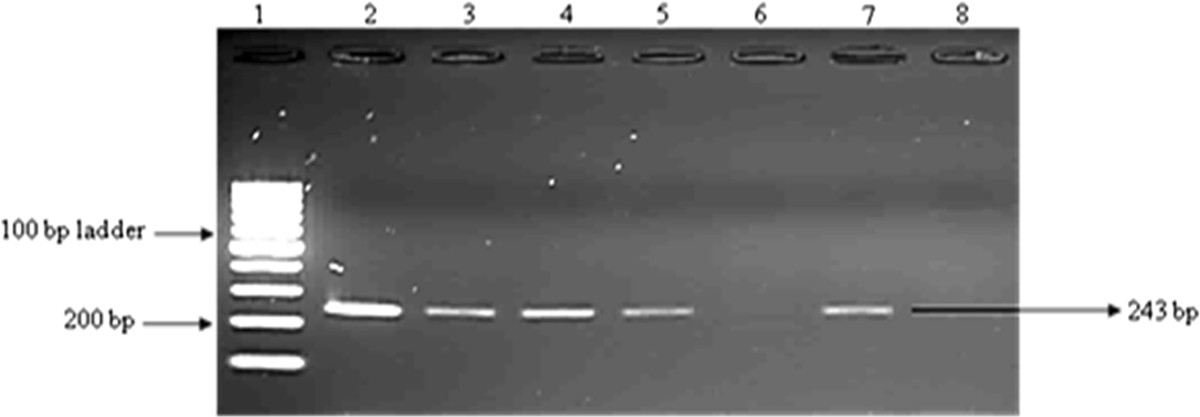
**Amplification of the** ***Kat G*** **gene in DNA samples.** Lane 1: 100 bp ladder; lane 2: positive control; lane 3–5 and 7: DNA isolates; lane 8: negative control.

**Figure 4:**
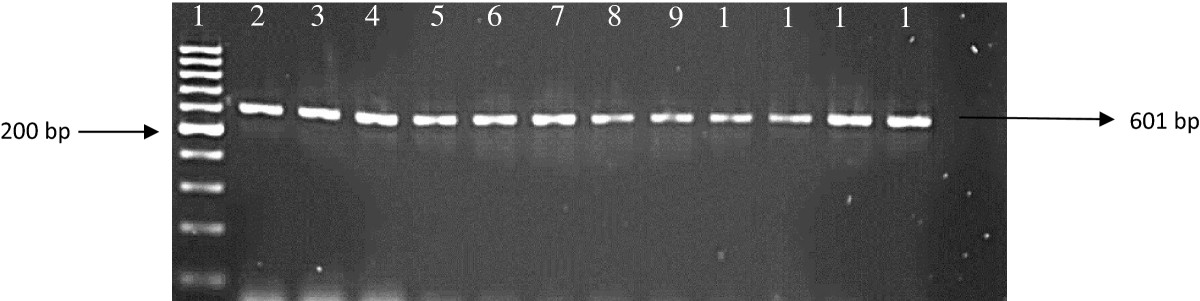
**Amplification of the** ***eis*** **gene in DNA samples.** Lane1: 100 bp ladder; lane 2: positive control; lane 3–13: DNA samples; lane 14: Negative control.

**Figure 5:**
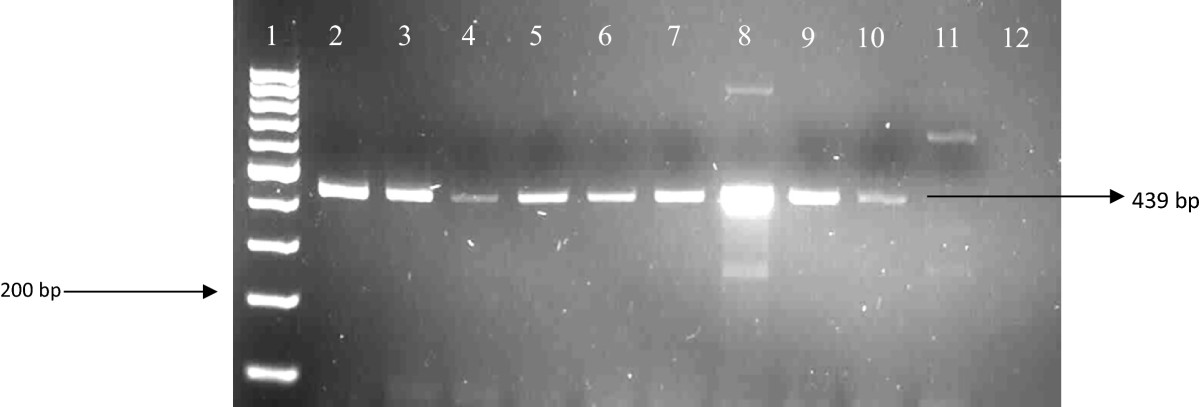
**Amplification of the** ***rrs*** **gene in DNA samples.** Lane 1: 100 bp ladder; lane 2: positive control; lane 3–10 DNA isolates; lane 12: negative control.

After amplification with the primers that amplify the *kat*G and *rpo*B genes, 157 isolates showed positive bands to rifampicin (*rpoB* gene) and 104 isolates showed positive bands to isoniazid (*katG* gene). The samples that did not show any bands were properly mixed and they were ran on 2% agarose gel again. We observed that all 190 amplicons showed bands which confer resistance to RIF and INH drugs (Figures 2 and 3). Amplification of the genes (*rrs* and *eis*), conferring resistance to second line regimen was observed in 39 isolates for *eis* gene and 89 isolates showed positive bands to injectable drugs (*rrs* gene) (Figures 4 and 5).

In our study we observed INH-resistant strains in 71.4% (100/140) with mutations at codon 315 on the analyzed isolates analyzed. The *katG* gene encodes the catalase peroxidise enzyme [35] and is present in variable regions of the MTBC genome and contains a repetitive DNA sequence. A study by Van Doom et al. [36] and Bokonyte et al. [37] showed high INH-resistant strains that had mutations on codon 315 (S to T) with St Petersburg of Russia (92%), Lithuania (85.7%) and Netherlands (89%). In our study 35.7% (50/140) had an amino acid change (S → N), this mutation was also seen in isolates from Spain [38], while 7.1% (10/140) (T → S) and 7.1% (10/140) (T → N) were also observed. This amino acid changes do not have much information reported on them. The high percentage of mutation at codon 315 and different substitutions demonstrates the importance of this codon in the development of INH resistance among strains from Port Elizabeth. A mutation at codon 293 seems to be rare and not much information on it has been given. In our samples twenty mutation 14.3% (20/140) were found at this position. We suggest that codon 293 is involved in the resistance mechanism in this isolates. A mutation 7.1% (10/140) (R → L) was also seen in the samples. Mutations from this position have been reported before by Haas et al. [39] from African strains. The very same mutations were reported by Musser et al. [40] who suggested an ancestral *kat G* (R) genotype for 127 isolates of *M. microti*, *M. bovis* and *M. africanum*[39]. Before the report of Walter et al. [41], the R mutation at position 463 was described for only *M. tuberculosis*[41]. In the *kat G* gene region, seven different mutations were observed, 35.7% (50/140), 14.3% (20/140) and 7.1% (10/140) five mutations (Table 3). Two isolates did not have any mutations suggesting that its not only the regions of DNA that were investigated in this study that confers resistance to IHN.

**Table 3:** **Frequency of mutations in** ***katG*** **gene codons 293, 315 and 463 in 140 INH-resistant strains of** ***M. tuberculosis*** **complex**

	*katG*gene mutation positions						
	N_293_ → G	S_293_ → G	S_315_ → T	S_315_ → N	T_315_ → N	T_315_ → S	R _463_ → L
No. of strains (%)	10 (7.1%)	10 (7.1%)	20 (14.3%)	50 (35.7%)	10 (7.1%)	10 (7.1%)	10 (7.1%)

N = Asparagine; G = Glycine; S = Serine; T = Threonine; R = Arginine; L = Leucine.

We also observed RIF-resistant strains in 99.5% (189/190) of the isolates with different mutations on the *rpoB* gene of 140 different strains were analyzed. The results obtained from this study were interesting; hence we observed other mutations that have not been reported previously (Y_42_ → D). In our study the prevalent mutation among the RIF-resistant isolates was at codon 42 (21.4%) and not much information has been published concerning this mutations. This mutation was followed by 14.3% of mutations on codon 52 (G → A), codons 87 (H → G); 92 (L → S); 441 (L → S); 450 (L → P) and 457 (showed 7.1% (10/140). Codon 531 is known to be a hot spot for *rpoB* gene mutations [42]. This mutation is reported in most studies, one of the studies in Taiwan reported 54.9% of mutations in codon 531 [42]. These mutations (at codon 531) were also observed in other countries such as Germany 71% [43], Italy 59% [44], Greece 56% [45], Japan 43% [46] and Mozambique 21% [47]. There were seven different mutations on this gene that were observed. The mutations are highlighted in (Table 4).

**Table 4:** **Frequency of mutations in** ***rpoB*** **gene codons 42, 52, 87, 92, 441, 450 and 457 in 140 RIF-resistant strains of** ***M. tuberculosis*** **complex**

	*rpoB*gene mutation positions						
	Y_42_ → D	G_52_ → A	H_87_ → G	L_92_ → S	V_441_ → G	L_450_ → S	L _457_ → P
No. of strains (%)	30 (21.4%)	20 (14.3%)	10 (7.1%)	10 (7.1%)	10 (7.1%)	10 (7.1%)	10 (7.1%)

Y = Tyrosine; D = Aspartic; G = Glycine; A = Alanine; H = Histidine; L = Leucine; S = Serine; V = Valine; P = Proline.

The *rrs* gene it consist of injectable anti-TB drugs such as amikacin (AMK), kanamycin (KAN) and capreomycin (CAP) [48]. Proper use of injectable drugs is critical to the effective treatment of MDR-TB and in prevention of XDR-TB [48]. Mycobacterium culture and susceptible testing in media either solid or liquid relies on conventional diagnosis of MTBC strains [49]. This method is not reliable for the detection of injectable drugs resistance [49]. AMK and KAN bind to the 16S rRNA in the 30S ribosomal subunit and inhibit protein synthesis [50] and CAP interferes with translation and inhibits phenylalanine synthesis in mycobacterial ribosome [51]. Mutations in the MTBC that prevents the binding of the injectable drugs to the targeted pathogen gene have been associated with resistance to the three injectable drugs [52, 53].

In our study among the 120 isolates sequenced, four types of mutation patterns were observed in the *rrs* gene region; S2170A, R2201G, K2202E and a deletion in position 2207. The most observed mutation within the region was an S → A 100% (120/120) substitution at position 2170 followed by 58.3% silent mutation (70/120) R → R, 66.7% (80/120) of K → E and a deletion 41.7% (5/12) at position 2207 (Table 5). Mutations shown in the table are more than the number of isolates that were sent for sequencing. This is because one isolate had more than one mutation in it which therefore increased the number of mutations that are seen in Table 5. Mutations associated with injectable drug resistance are under studied in comparison with mutations associated with first-line drugs [54]. Most studies have reported on C1143G and T1521C in the *rrs* gene. In our study, these mutations were not found in the isolates studied and have not been described as conferring resistance [55]. Studies by Maus et al. [56] and Krüüner et al. [57] has reported on mutations in the 500 *rrs* region A514C and C417T, even these mutations were not found in our study.

**Table 5:** **Frequency of mutations in** ***rrs*** **gene showing nucleotide change in 120** ***rrs*** **-resistant strains of** ***M. tuberculosis*** **complex**

	***rrs***gene mutation positions			
	S_2169,70_ → A	R_2201_ → R	K_2202_ → E	Deletion_2207_
No. of strains (%)	120 (100%)	70 (58.3%)	80 (66.7%)	50 (41.7%)

S = Serine; A = Alanine; R = Arginine; K = Lysine; E = Glutamic acid.

## Conclusions

The results obtained from this study show a high prevalence of MTBC among Eastern Cape population. Of noteworthy is the fact that women at their reproductive years are mostly infected and this could lead to a vicious cycle, hence women are exposed to a lot of people. The study also revealed a high prevalence of MDR amongst the Eastern Cape population.

## Authors’ original submitted files for images

Below are the links to the authors’ original submitted files for images. Authors’ original file for figure 1 Authors’ original file for figure 2 Authors’ original file for figure 3 Authors’ original file for figure 4 Authors’ original file for figure 5

## Abbreviations

AIDS: Acquired Immunodeficiency Syndrome
DNA: Deoxy-Ribonucleic Acid
DST: Drug Susceptibility Testing
EC: Eastern Cape
Eis: Streptomyces Coelicolor
EMB: Ethambutol
GyrA: Gyrase
HIV: human Immune Virus
INH: Isoniazid
KatG: Catalase-Peroxidase
MDR: Multi-Drug Resistant
MPB64: Immunogenic protein
MTBC: *Mycobacterium Tuberculosis* Complex
NaCl: Sodium Chloride
NaOH: Sodium Hydroxide
PCR: Polymerase Chain Reaction
PZN: Pyrazinamide
RIF: Rifampicin
*rpo*B: the gene that encodes the β subunit of bacterial RNA polymerase
Rrs: 16S rRNA
TB: Tuberculosis
WHO: World Health Organization
XDR: Extensively Drug Resistant
ZN: Ziehl-Neelsen.
