# Supplementary figures and images for: Molecular detection and characterization of resistant genes in Mycobacterium tuberculosis complex from DNA isolated from tuberculosis patients in the Eastern Cape province South Africa
Source: BMC Infect Dis. 2014 Sep 4;14:479. doi: 10.1186/1471-2334-14-479 (PMC4161913; doi:10.1186/1471-2334-14-479)

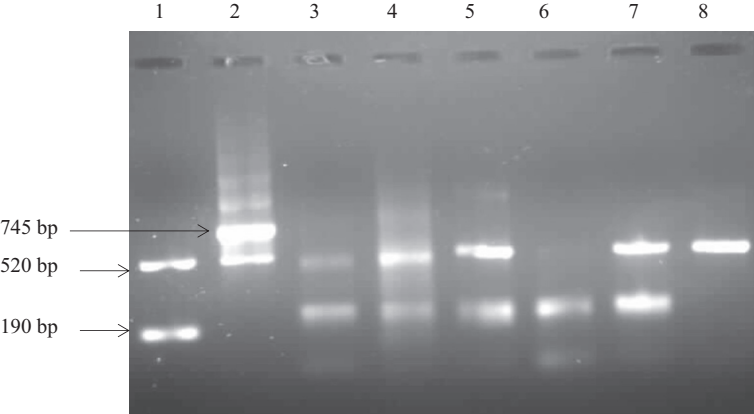

Supplement: Supplementary file 1 — Authors’ original file for figure 1 [file 12879_2014_3784_MOESM1_ESM.pdf]

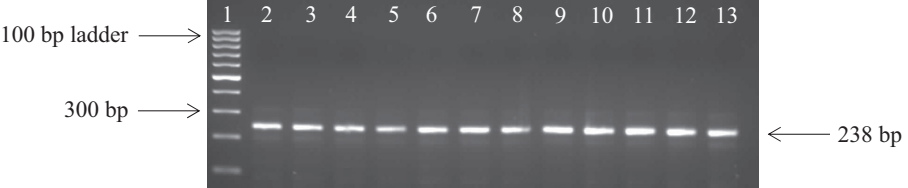

Supplement: Supplementary file 2 — Authors’ original file for figure 2 [file 12879_2014_3784_MOESM2_ESM.pdf]

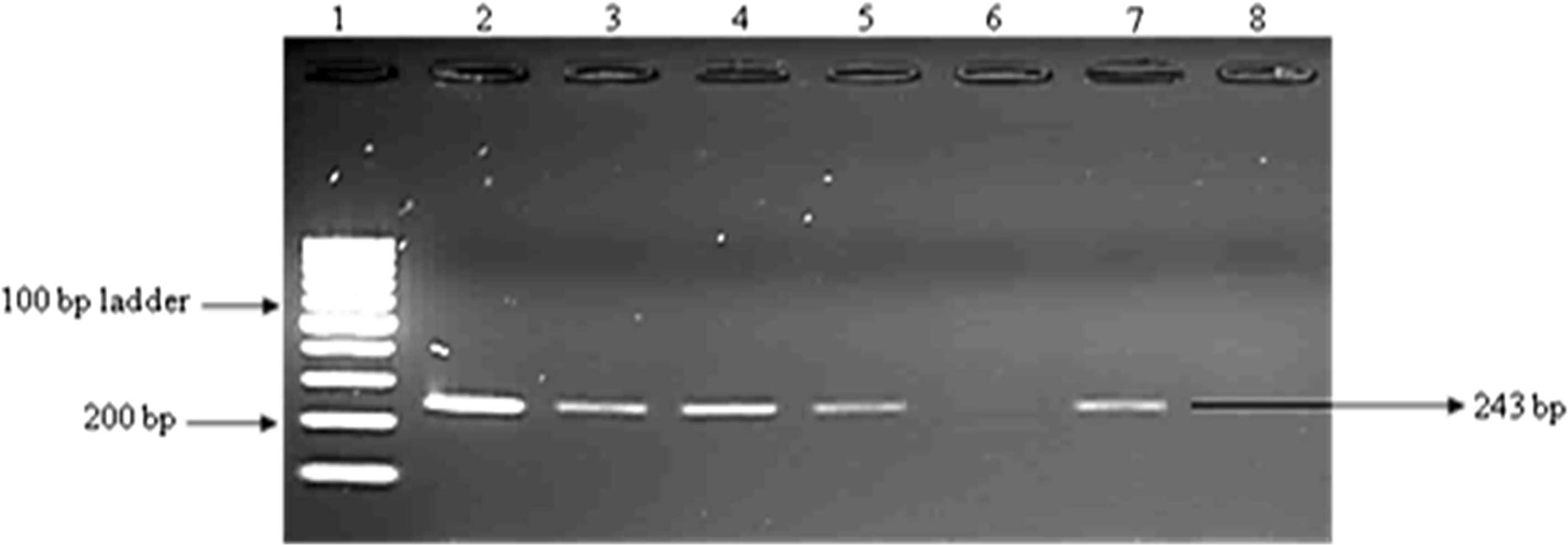

Supplement: Supplementary file 3 — Authors’ original file for figure 3 [file 12879_2014_3784_MOESM3_ESM.tif]

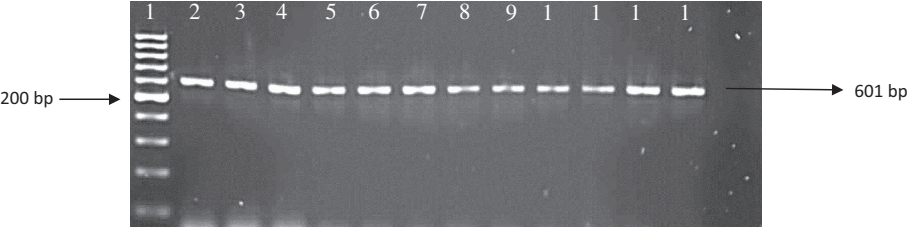

Supplement: Supplementary file 4 — Authors’ original file for figure 4 [file 12879_2014_3784_MOESM4_ESM.pdf]

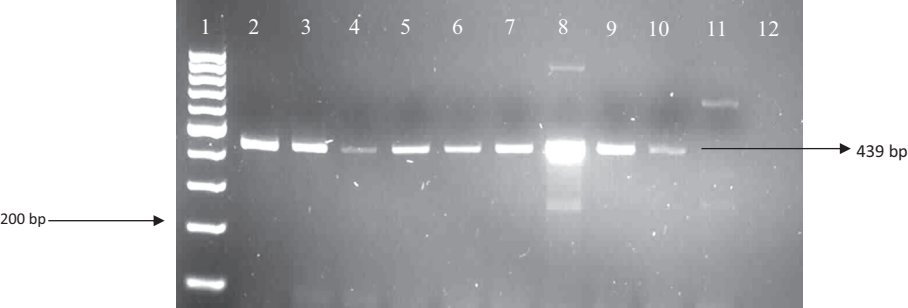

Supplement: Supplementary file 5 — Authors’ original file for figure 5 [file 12879_2014_3784_MOESM5_ESM.pdf]
